# Analyzing a Community-based Coalition’s Efforts to Reduce Health Disparities and the Risk for Chronic Disease in Kansas City, Missouri

**Published:** 2007-06-15

**Authors:** Vicki L Collie-Akers, Stephen B Fawcett, Jerry A Schultz, Valorie Carson, John Cyprus, Joseph E Pierle

**Affiliations:** Work Group for Community Health and Development; University of Kansas, Lawrence, Kan; University of Kansas, Lawrence, Kan; University of Kansas, Lawrence, Kan; Kansas City-Chronic Disease Coalition and Missouri Primary Care Association, Kansas City, Mo; Kansas City-Chronic Disease Coalition and Missouri Primary Care Association, Kansas City, Mo

## Abstract

**Background:**

Although it is well known that racial and ethnic minorities in the United States have a higher prevalence of chronic diseases and a higher rate of related deaths than the overall U.S. population, less is understood about how to create conditions that will reduce these disparities.

**Context:**

We examined the effectiveness of a collaborative community initiative ― the Kansas City-Chronic Disease Coalition ― as a catalyst for community changes designed to reduce the risk for cardiovascular diseases and diabetes among African Americans and Hispanics in Kansas City, Missouri.

**Methods:**

Using an empirical case study design, we documented and analyzed community changes (i.e., new or modified programs, policies, or practices) facilitated by the coalition, information that may be useful later in determining the extent to which these changes may contribute to a reduced risk for adverse health outcomes among members of the target population. We also used interviews with key partners to identify factors that may be critical to the coalition's success.

**Results:**

We found that the coalition facilitated 321 community changes from October 2001 through December 2004. Of these changes, 75% were designed to reduce residents' risk for both cardiovascular disease and diabetes, 56% targeted primarily African Americans, and 56% were ongoing. The most common of several strategies was to provide health-related information to or enhance the health-related skills of residents (38%).

**Conclusion:**

Results suggest that the coalition's actions were responsible for numerous community changes and that certain factors such as hiring community mobilizers and providing financial support to nontraditional partners may have accelerated the rate at which these changes were made. In addition, our analysis of the distribution of changes by various parameters (e.g., by goal, target population, and duration) may be useful in predicting future population-level health improvement.

## Background

Throughout its history, the United States has had health disparities associated with race and ethnicity. The gap in standardized mortality rates between African Americans and whites remained largely unchanged from 1960 to 2000 (1). In 2003, the Centers for Disease Control and Prevention (CDC) reported that the risk for diabetes was 1.6 times higher among African Americans and 1.5 times higher among Hispanics than it was among whites (2). CDC also reported that, in 2001, the rate of death from heart disease was 31% higher among African Americans than among whites (3).

Health disparities have multiple and interrelated causes, including discriminatory policies and practices by some primary care providers and hospitals (4). For example, a study of Medicare users published in 1996 indicated that, on average, African American users received fewer mammograms and immunizations than white users (5). Income inequality may also help explain health disparities, as Kawachi et al (6) suggested by showing that the risk for heart disease among low-income African Americans was closer to that among low-income whites than that among middle- or higher-income African Americans. Other factors that may contribute to racial and ethnic disparities in health include differences in health risks associated with environmental and cultural factors (7).

One challenge to addressing the multiple and interrelated causes of health disparities is that it requires collaboration among many different individuals and organizations (8). Research results suggest, however, that approaches such as improving the primary health care system can help reduce health disparities, and that the use of registries, evidence-based standards of care, patient education, and coordinated care can all contribute to improved primary care (9). CDC established the Racial and Ethnic Approaches to Community Health 2010 (REACH 2010) initiative to promote the use of community collaborations as a strategy to connect members of targeted communities with efforts to reduce health disparities related to cardiovascular diseases, diabetes, HIV/AIDS, infant mortality, breast and cervical cancer, and child and adult immunizations. A recent REACH project used community health advisors to improve the health outcomes of community residents (10). Other types of research conducted with the support of the REACH 2010 initiative include descriptions of participatory approaches used to engage community members as part of coalitions or partnerships (11,12), the collection of data on disease prevalence (13), a description of approaches used to engage women of color in REACH initiatives (14), and a description of minority health based on national REACH 2010 survey data (15).

The purpose of this study was to document and analyze the community changes facilitated by a REACH 2010 initiative of the Kansas City-Chronic Disease Coalition (KC-CDC). These changes are intermediate outcomes related to KC-CDC's long-term goal of reducing the risk for cardiovascular disease and diabetes among African Americans and Hispanics in an 11-zip-code target area of Kansas City, Missouri. In the study, we sought to answer two research questions: 1) To what extent did the KC-CDC serve as a catalyst for community changes? and 2) What factors or mechanisms were associated with increases or decreases in the rate at which these changes occurred?

In addition, the data we collected for this study may be useful in answering a third, long-range research question: How do the community changes facilitated by the KC-CDC contribute to changes in population-level health outcomes? 

## Context

In 2000, two separate health assessments conducted in the Kansas City metropolitan area helped to identify local health disparities. The results of a health assessment commissioned by the United Auto Workers-Ford Community Health Care Initiative (UAW-Ford CHCI) and conducted by the Lewin Group showed significant health disparities between African Americans and Hispanics and other residents of the greater Kansas City area (16). The results of a similar assessment by the Kansas City, Missouri, Health Department showed that Kansas City residents who were racial or ethnic minorities had a life expectancy 11 years shorter than white residents in the same area and that, compared with white residents, African American residents were 2.5 times more likely to die of diabetes and 1.5 times more likely to die of cardiovascular diseases, and Hispanics were 1.5 times more likely to die of diabetes (17).

In 2000, CDC awarded the Missouri Primary Care Association (MPCA) a REACH 2010 contract to develop a community action plan to address health disparities experienced by minority populations in Kansas City, Missouri. In the course of developing the plan, MPCA collaborated with multiple partners, including representatives from five health centers, the UAW-Ford CHCI, several neighborhood organizations, and the local health department. The KC-CDC was also established during this planning effort. In 2001, MPCA and KC-CDC applied for and received a cooperative agreement award from CDC to implement their community action plan. The coalition initiated a program called Pick Six, in which coalition partners were asked to identify six community changes that they could implement. From October 2001 through December 2004, coalition partners were given subcontracts to implement the community changes that they had identified in the action plan. The partners consisted of 5 community health centers, 24 neighborhood associations, 24 faith organizations, and several other public and private organizations.

In accordance with the findings in the Kansas City Health Department's report (17), the coalition focused on two minority populations at high risk for cardiovascular disease and diabetes: African Americans and Hispanics. As a result, the coalition targeted an economically disadvantaged area of central Kansas City with the highest density of African Americans and Hispanics. According to the 2000 United States Census estimates (18), 159,580 people resided in the targeted area. Of this population, 57% (91,088) were African American, 8.5% (13,515) were Hispanic, and 24% (38,385) had household incomes below the poverty line. Specific objectives established by the planning group included increasing the percentage of adult residents who 1) could identify a primary care provider, 2) reported engaging in regular physical activity, 3) reported consuming five or more servings of fruits and vegetables per day, and 4) reported having had an HbA1c test in the previous year. (This study did not address the coalition's success in meeting these objectives.)

Consistent with the Institute of Medicine's Framework for Collaborative Public Health Action in Communities (19,20), the KC-CDC used a logic model with five interrelated phases: 1) collaborative planning and capacity building, 2) targeted action and intervention, 3) community and system changes, 4) widespread behavior change, and 5) improvements in community health outcomes. This report summarizes the KC-CDC's efforts in the first three of these phases.

During the collaborative planning and capacity-building phase, coalition partners assessed the extent of racial and ethnic disparities in health among Kansas City-area residents and developed a plan for reducing these disparities. During the second phase, targeted action and intervention, they engaged in advocacy work and other activities necessary for the institution of a new or modified program, policy, or practice. During the third phase, they actually implemented these new or modified programs, policies, or practices. During the fourth phase, we intend to determine whether the percentage of residents who engage in various healthful behaviors increases following the implementation of these changes. And during the fifth phase, we intend to determine whether the incidence and prevalence of diabetes, cardiovascular disease, and their related complications decrease among members of the target populations (21).

Throughout all phases of the effort, the University of Kansas Work Group for Community Health and Development (KU Work Group) served as the scientific partner of KC-CDC. Using a process of community-based participatory research (CBPR) (22), researchers, coalition staff, and members of the coalition worked together to establish research questions and a measurement system to answer those questions. Community partners assumed responsibility for documenting instances of community or system change. The KU Work Group ensured the quality of collected data, provided reports to the group, including an analysis of how these changes might contribute to achieving KC-CDC's community-determined goals and objectives, and prompted a dialogue among coalition members about possible adjustments in the work of the KC-CDC. This research was approved by the University of Kansas Human Subjects Committee (approval number 14958).

## Methods

We used an empirical case study design as described by Yin (23).

To document and analyze KC-CDC's effectiveness in producing the intermediate outcomes of community or system changes, we used an online documentation and support system (24,25). KC-CDC partners and staff members were trained to use the documentation system to record discrete events and activities in their community. They described these events and activities in a narrative format and then coded them using definitions and scoring instructions to differentiate four types of events or activities: 1) community changes, 2) community actions, 3) the production of "planning products," and 4) other events. We analyzed the distribution of events or activities coded as "community changes" by their targeted goal, the sector of the community in which they occurred, the behavioral change strategy they used, and their duration. These data may be useful in future analyses of correlations between specific types of community changes and reductions in risk for cardiovascular disease and diabetes.

A plurality of the events or activities were coded as community changes, which we defined as new or modified programs, policies, or practices facilitated by the coalition or its partners and related to its mission of reducing racial and ethnic health disparities in the Kansas City area (26). To be coded as a community change, an event must have 1) been an instance of a new or modified program, policy, or practice; 2) already occurred; 3) included community residents who were not members of the coalition; 4) been related to the initiative's goals and objectives; and 5) been facilitated by the coalition members or partners.

Coalition partners and staff members received documentation training that included written and oral descriptions of coding definitions, examples of community changes, opportunities to practice scoring, and feedback on the accuracy of their scoring. Local documenters who were part of the coalition were asked to enter community changes as they occurred or, at a minimum, during contract reporting times. In some instances, members of the coalition staff or the KU Work Group staff recorded events on behalf of a coalition partner. One member of the KU Work Group provided primary coding for all of the 729 documented entries, and a secondary coder, another member at the KU Work Group, independently coded 89 (12%) of the entries. By dividing the number of entries coded the same by both coders (77) by the total number of entries coded by both (89), we found an 86.5% interobserver agreement between the primary and the secondary coder. The *κ*score for the reliability between observers was 0.79.

During January and February 2005, we also conducted qualitative interviews with 12 key partners, including leaders of neighborhood and faith organizations, members of the private sector, and members of the community health center staff and coalition staff. Using a standard interview protocol, we asked interviewees to describe the strengths and challenges of the work they do and their key achievements and events. We then reviewed recordings of these interviews to distill common themes.

### Intervention 

The KC-CDC provided several support functions to educate coalition partners and help them implement community changes. These included using a newsletter and coalition meetings to educate partners about cardiovascular disease and diabetes and the risk factors for them; providing information resources to all partners; employing community mobilizers to help partners plan and implement changes; documenting the coalition's accomplishments and using this information to modify future community change efforts; establishing clear vision and mission statements and a framework for action; developing an action plan that allowed prospective partner organizations to see how they could contribute to the coalition efforts; promoting the sharing of resources among coalition partners; providing subcontractors to help partners implement the community action plan; and using recognition ceremonies to celebrate the accomplishment of partners. The results of these coalition activities (which we will treat as independent variables in subsequent analyses) were community and system changes (which we will treat as intermediate outcomes in subsequent analyses).

## Results

Of the 729 events or activities facilitated by the KC-CDC (data not shown), we coded 321 as being community changes. The Figure displays the cumulative number of community changes facilitated by the coalition as they occurred over time. We divided these community changes into three categories: new programs, new policies, and new practices. An example of a new program was a Friday night physical activity program at Beacon Hills Church of the Nazarene (faith community sector) that began in November 2003; an example of a new policy was a smoke-free workplace ordinance passed by the Kansas City, Missouri, City Council on November 23, 2004 (government sector); and an example of a new practice was the UAW-Ford Community Healthcare Initiative (private sector), which led to an increase in the number of primary care physicians providing feedback reports on the care provided to patients with diabetes (from 300 in September 2003 to 625 in December 2004).

The Figure , which displays a cumulative record of community changes with an overlay of critical events, shows that several factors were associated with marked increases or decreases in the rate at which the changes were instituted. These factors included the hiring of a project manager; the hiring (and later the departure) of a community mobilizer to help implement the action plan; and the use of annual subcontracts, or minigrants, making targeted resources available to neighborhood and faith organizations. The rate at which community changes occurred decelerated after the end of KC-CDC's program to distribute minigrants to neighborhoods.

**Figure:**
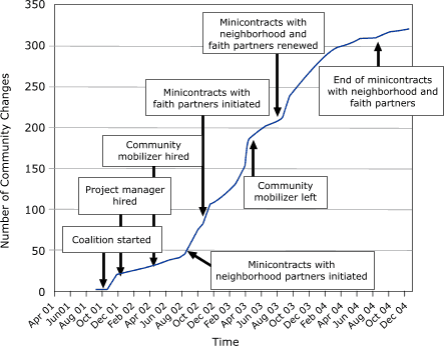
Cumulative number of community changes facilitated by the Kansas City-Chronic Disease Coalition from its inception through December 2004 (N = 321).

**Table:** 

We analyzed the 321 documented community changes facilitated by the KC-CDC so that researchers may later determine how they contributed to the longer-term outcomes targeted by the initiative (i.e., to population-level changes in health-related behavior and in actual health outcomes). To do so, we coded each documented community change by six parameters: 1) the health condition targeted, 2) the duration of the change or event, 3) the behavioral change strategy used, 4) the community sector targeted, 5) the population targeted, and 6) the risk factor addressed (Table). We found that 75% of the community changes facilitated by the KC-CDC were designed to reduce residents' risk for both cardiovascular disease and diabetes; that 28% were one-time events, 15% occurred more than once, and 56% were ongoing activities; that the two most frequently used behavioral change strategies were providing information and enhancing skills (38%) and modifying access, barriers, and opportunities (27%); that the three community sectors in which changes occurred most frequently were neighborhood networks (35%), health care providers (21%), and faith organizations (20%); that African Americans were the primary targets of 56% of the community changes and Hispanics the primary targets of 13%; and that the most frequently targeted risk factors were poor nutrition (26%), physical inactivity (17%), and lack of access to quality care (17%), with 20% of the community changes targeting multiple risk factors.

## Interpretation

We found that the KC-CDC facilitated 321 community changes in 38 months, with the bulk of these changes occurring in the first 2 years of the coalition's activities. We identified two factors as being critical to the coalition's success in bringing about these changes. The first was the development of four key planning products that guided the coalition's efforts: a vision statement, a mission statement, a logic model, and an action plan. The second key factor was the hiring of a community mobilizer to help implement the action plan, develop partnerships, and provide technical assistance. The departure of the first mobilizer was correlated with a temporary decrease in the rate of community changes, suggesting that her engagement was important to the coalition's ability to facilitate change. The 12 key partners we interviewed indicated that the leadership of the project manager was also critical to the success of the coalition.

The coalition facilitated community changes designed to reduce residents' risk for two primary disease processes: cardiovascular diseases and diabetes; 43% of the coalition's activities focused on reducing the prevalence of unhealthy diets and physical inactivity, behavioral risk factors for both disease processes. We also found that 56% of the community changes were sustained over time and that many used multiple behavioral change strategies (data not shown).

During the period that we studied, 56% of the community changes facilitated by the coalition targeted primarily African Americans, and 13% targeted primarily Hispanics. Although the percentage of changes targeting primarily Hispanics exceeded the percentage of Hispanics in the target area, coalition members concluded that there were too few community changes in Hispanic neighborhoods to significantly affect population-level health outcomes, and therefore they identified additional activities to increase the number of community changes targeting Hispanics.

We identified four particular strengths of the KC-CDC. First, its strategy of engaging neighborhood and faith groups by making resources available to them seemed to work well, given that these groups were responsible for 55% of the changes identified by coalition partners as beneficial to the community yet received only 16% coalition's resources (data not shown). Second, its "multisector approach" was successful in engaging diverse parts of the community representing different interests and with access to different resources. Third, by engaging different parts of the community, the coalition created opportunities for partners to change conditions at multiple levels. Finally, by engaging multiple partners, the coalition was able to help facilitate ongoing programs, policies, and practices related to different risk factors, and because these activities were implemented by the coalition's partners, they are more likely to be maintained even if the coalition itself is not.

This study has two primary limitations. First, its case study design will preclude us or other researchers from using study results to determine whether any of the community changes will be the cause of any eventual changes in population-level behavioral practices or health outcomes (27). However, although the results from a case study are not expected to show definitive causal relationships, they can be used to build hypotheses that may be useful in later research and action (23); in this case, the results of our study may help show how the coalition processes implemented by the KC-CDC created conditions (community changes) that may contribute to subsequent changes in the health of community residents. Its second notable limitation is that most of the data in this study were based on reports by coalition partners and were not independently verified by objective observers.

We suggest that future research concerning the effectiveness of community efforts to reduce health disparities should focus on two areas. First, it should use more rigorous experimental methods to examine the effectiveness of coalition processes such as the development of an action plan and the use of a community mobilizer. For instance, researchers could use multiple time-series designs, with staggered introduction of key processes across coalitions, to examine the extent to which these processes are associated with an increase in the rate of community changes. Second, future research should use methods such as the interrupted time-series design to examine the extent to which the activities of community coalitions are associated with actual improvement in the health of the targeted population. A time-series design can reduce many threats to the internal validity of study data and still allow for the study of multicomponent community interventions over time without the cost or other problems associated with a randomized control trial (28).

Despite its limitations, our study has a number of strengths, including the use of systematic methods to document community changes brought about by a community health initiative and to analyze these changes by key aspects such as their goal, duration, and priority population. Such descriptive information will be useful in future assessments of the coalition's effectiveness in improving the health-related behaviors and health outcomes of the target populations. In addition, the study's CBPR framework allowed coalition members to participate fully in naming and framing the problem, collecting data, and understanding what factors were critical to the success of the effort to produce community changes (22,25).

The health and well-being of a community's residents are key indicators of the functioning and social equality of that community. In any society, disparities in health outcomes among subgroups are a marker of social injustice (29). The efforts of the KC-CDC represent an attempt to eliminate health disparities among Kansas City residents by creating beneficial community changes in disadvantaged, predominately minority neighborhoods. Early findings suggest that the coalition has been effective as a catalyst for change by helping to create conditions that may improve the health behaviors and eventually the health outcomes of community residents. We hope that the preliminary analysis described here can contribute to a better understanding of the processes by which community partnerships promote health for all residents and, we hope, to an improvement in those processes.

## Figures and Tables

**Table:** Percentage Distribution, by Selected Parameters, of 321 Community Changes Documented in Analysis of Effectiveness of Kansas City-Chronic Disease Coalition, 2001–2004

**Parameter**	%
**Goal targeted**	
Cardiovascular disease and diabetes	75
Diabetes	13
Cardiovascular disease	6
General health care access/disparities	5
**Duration of change or event**	
Ongoing	56
One-time event	28
More than one-time event	15
**Strategy used**	
Providing information and enhancing skills	38
Modifying access, barriers, and opportunities	27
Changing the consequences	14
Enhancing services and support	10
Modifying policies	9
Other	1
**Community sector targeted**	
Neighborhood networks	35
Health care providers and organizations	21
Faith organizations	20
Private sector	7
Schools and education	5
Human services	3
Local government	3
Media	2
Other	2
**Population targeted**	
Primarily African Americans	56
Multiple racial/ethnic groups	18
Primarily Hispanics	13
Primarily African Americans and Hispanics	7
Other	6
**Risk factor targeted**	
Poor nutrition	26
Multiple risk factors	20
Other	19
Lack of access to quality care	17
Physical inactivity	17
Tobacco use	1
